# Supplementary material for: Cell-specific alterations in Pitx1 regulatory landscape activation caused by the loss of a single enhancer
Source: Nat Commun. 2021 Dec 13;12:7235. doi: 10.1038/s41467-021-27492-1 (PMC8668926; doi:10.1038/s41467-021-27492-1)
Supplement: Supplementary file 1 — Supplementary Information [file 41467_2021_27492_MOESM1_ESM.pdf]

Cell-specific alterations in *Pitx1* regulatory landscape activation caused by the loss of a single enhancer

Raquel Rouco[1,2*], Olimpia Bompadre[1,2*], Antonella Rauseo[1,2], Olivier Fazio[3], Rodrigue Peraldi[1,2,4], Fabrizio Thorel[3], Guillaume Andrey[1,2]

Supplementary Info

Supplementary figure S1

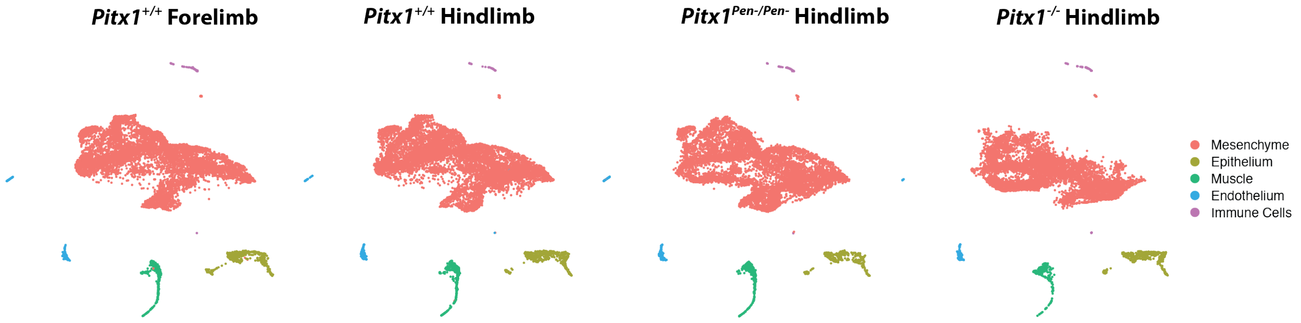

Figure S1: UMAP distribution of all cell clusters (Mesenchyme, Epithelium, Muscle, Endothelium and Immune Cells) in all datasets.

Supplementary figure S2

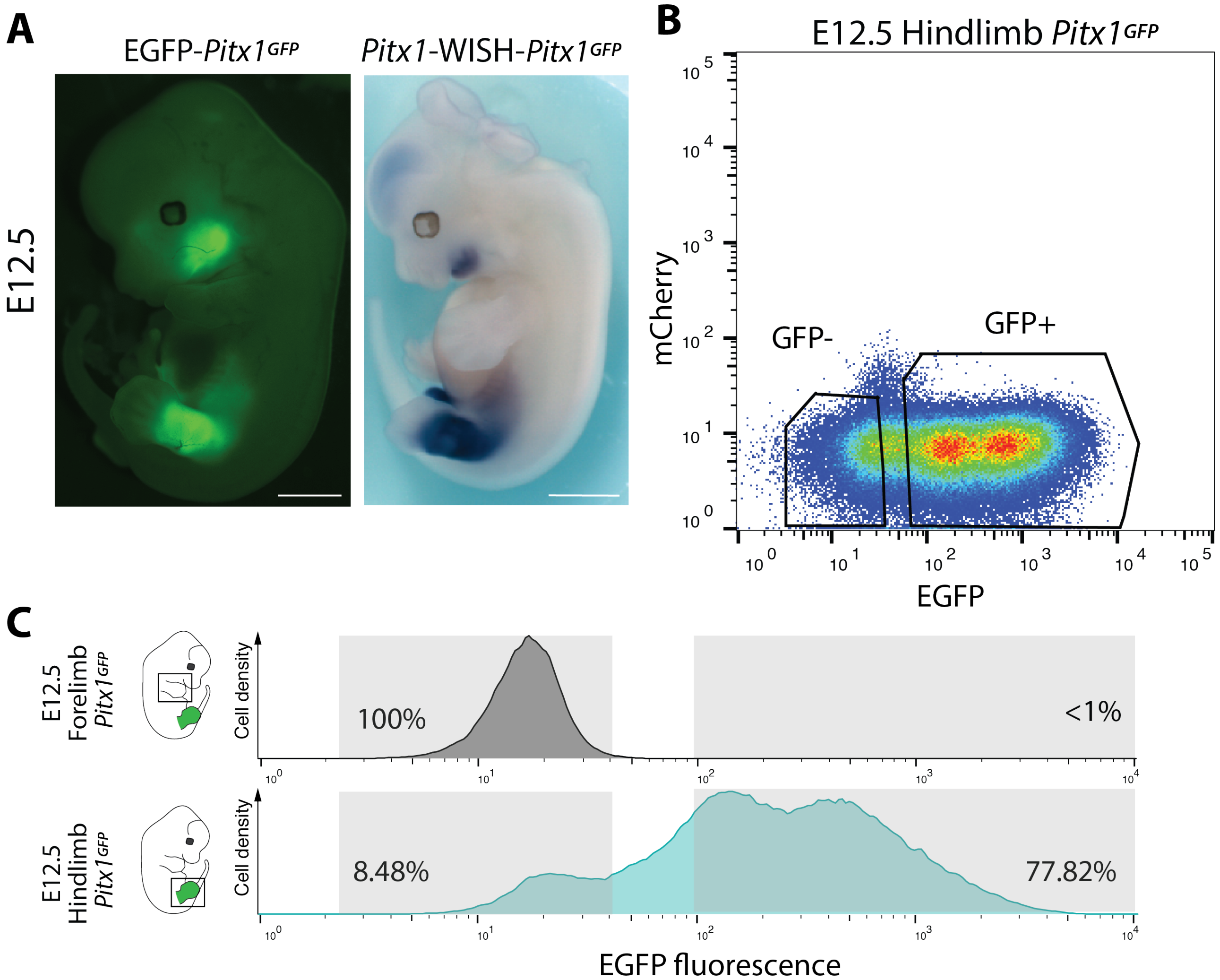

Figure S2: FACS sorting of *Pitx1*^{GFP} cells. A. EGFP fluorescence and *Pitx1* WISH of an E12.5 *Pitx1*^{GFP} embryo (N=3), scale bar=2mm. **B.** Overview of FACS gating of a E12.5 *Pitx1*^{GFP} hindlimb single cell preparation (mCherry signal on the y-axis and EGFP signal on the x-axis). **C.** Histogram of EGFP signal of *Pitx1*^{GFP} forelimb cells (upper track), here used to delimit the gating of GFP- cells, and *Pitx1*^{GFP} hindlimb cells (lower track).

Supplementary figure S3

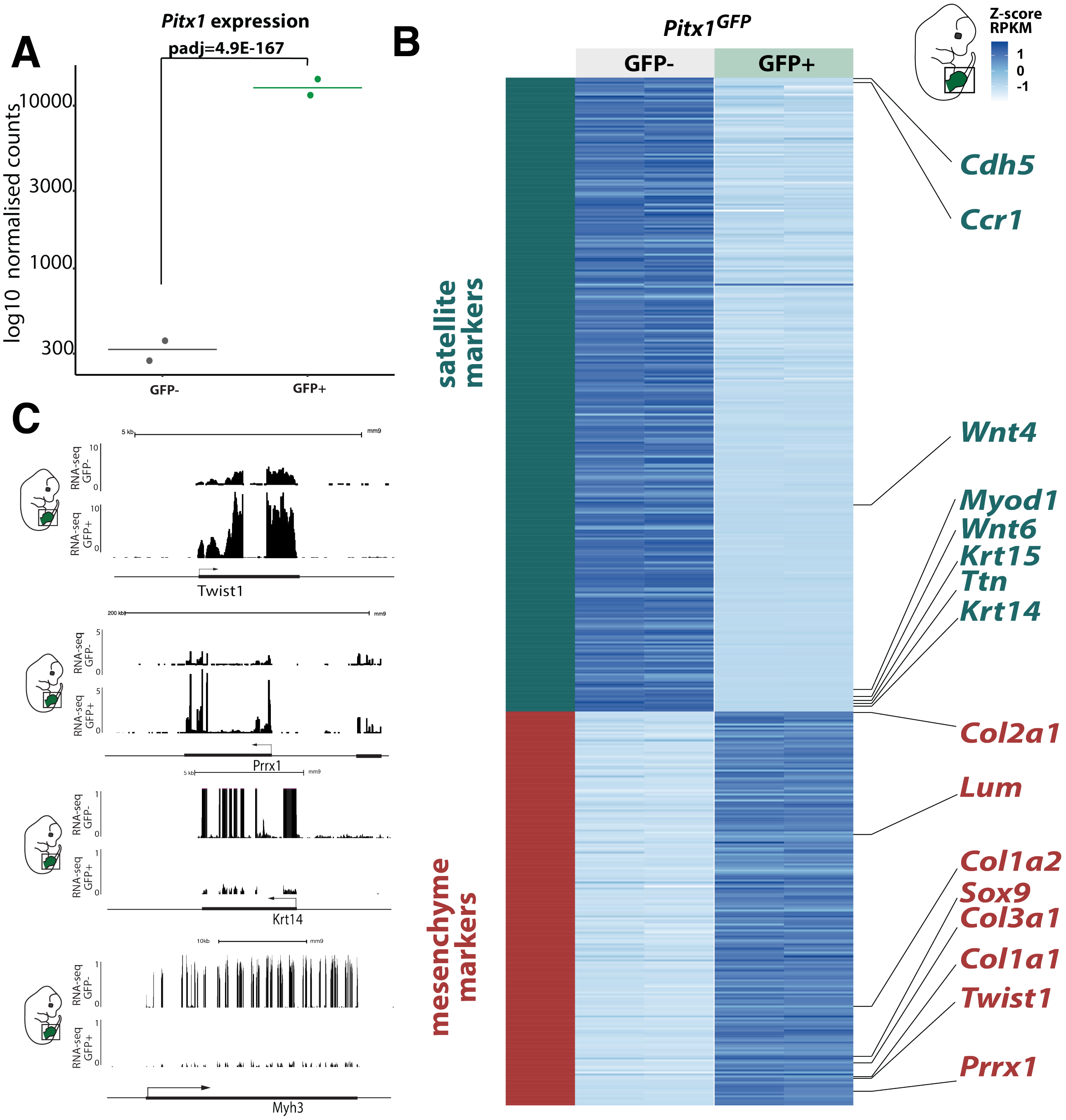

Figure S3: A. *Pitx1* normalized RNA-seq counts in GFP- and GFP+ *Pitx1*^{GFP} hindlimb cells. Averages are represented by a horizontal bar. Adjusted p-value (padj) of differential gene expression are computed using the Wald-test and Benjamini-Hochberg multiple testing correction as implemented in the Deseq2 tool (Supplementary Dataset S3 and Source Data). **B.** Heatmap of differentially expressed mesenchymal (red) and satellite (darkgreen) marker genes in GFP+ and GFP- cells of *Pitx1*^{GFP} hindlimbs. Note that GFP+ cells are enriched for condensating cells (*Sox9*, *Col2a1),* connective tissue (*Col1a1, Col2a1, Col3a1*) and mesenchymal patterning genes (*Shox2*, *Twist1, Prrx1*). GFP- cells are enriched for muscle (*Myod1*, *Ttn*), epithelial (*Wnt6*, Wnt4, *Krt14*, *Krt5*), immune (*Ccr1*) and endothelial cells (*Cdh5*). **C.** RNA-seq tracks of the *Twist1*/*Prrx1* mesenchymal, *Krt14* epithelium and *Myh3* muscle marker loci in GFP- and GFP+ *Pitx1*^{GFP} hindlimb cells. Note that in GFP- cells mesenchymal genes are lowly expressed, indicating that some GFP- cells are of mesenchymal origin. Oppositely, in GFP+ cells a slight expression of epithelium (*Krt14*) and muscle (*Myh3*) markers is present indicating that some epithelium and muscle cells express *GFP* and *Pitx1*.

Supplementary figure S4

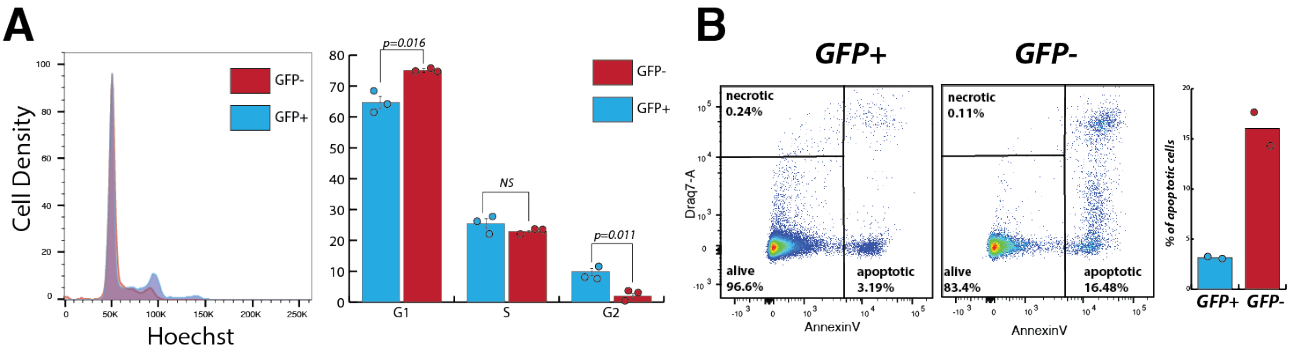

Figure S4: A. FACS proliferation assay using Hoechst staining of micro-dissected *Pitx1*^{GFP} hindlimb cells. Note that GFP- cells are depleted of G2-state cells and enriched for G1 cells (N=3). Error bars represent the standard deviation, error bar centers are the mean of the replicates and p-values (p) are calculated with a two sided t-test (See Source Data). **B.** FACS apoptosis assay using AnnexinV in micro-dissected *Pitx1*^{GFP} hindlimb cells. Note that GFP- cells contain more apoptotic cells than GFP+ cells (N=2).

Supplementary figure S5

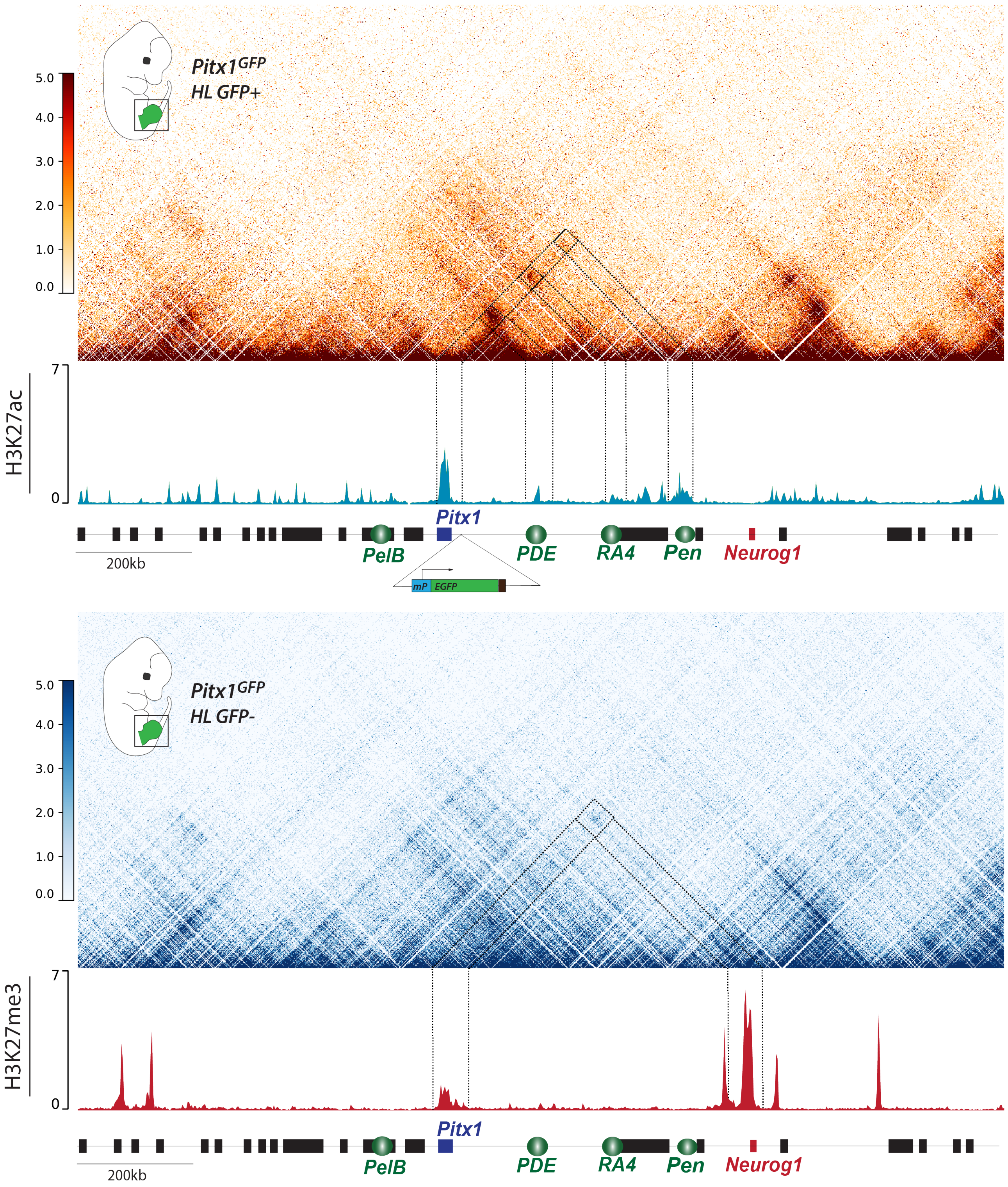

Figure S5: A. Above:1kb resolution C-HiC map of *Pitx1*^{GFP} GFP+ cells. Below: H3K27ac ChIP-seq track in GFP+ cells. Note alignment between C-HiC interactions and acetylation peaks. Darker red bins represent more frequent contacts as represented by scaled bar on the left. **B.** Above: 1kb resolution C-HiC resolution map of *Pitx1*^{GFP} GFP- cells aligned with, below, H3K27me3 ChIP of E11.5 entire hindlimb preparation (Kragesteen, et al., 2018). Note C-HiC contacts between *Pitx1* and *Neurog1* match enrichment of H3K27me3. Darker blue bins represent more frequent contacts as represented by scaled bar on the left.

Supplementary figure S6

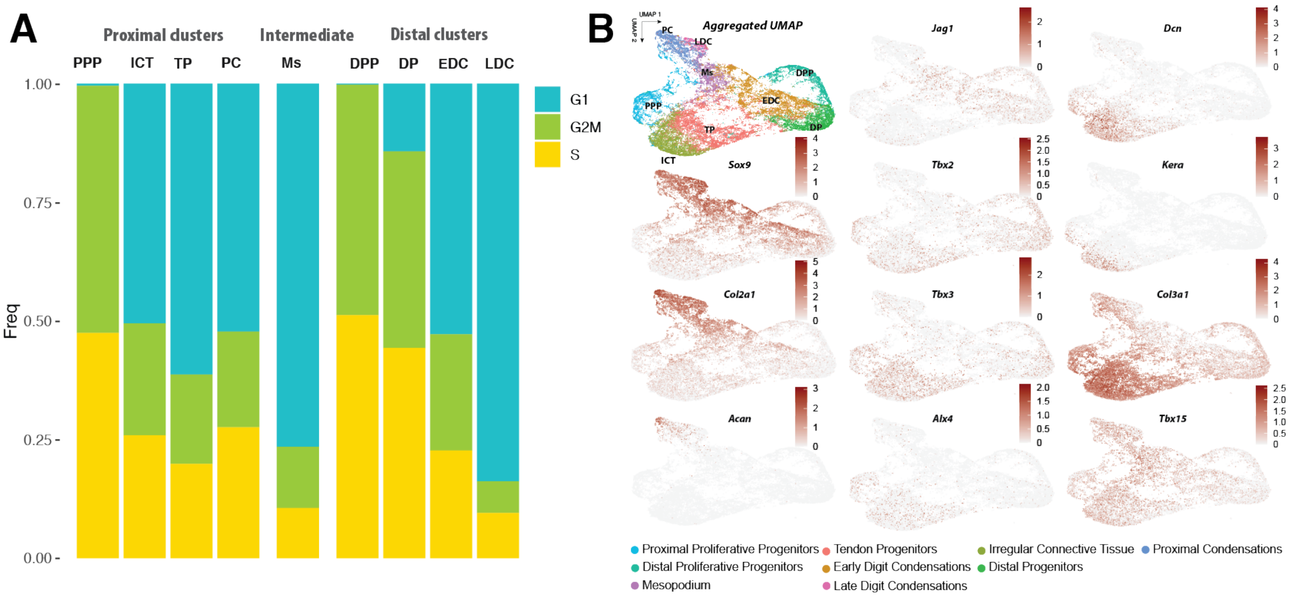

Figure S6: A. Analysis of cell cycle phase distribution in the different mesenchymal clusters. **B.** UMAP distribution of marker genes for chondrocytic lineage (*Sox9*, *Col2a1*, *Acan*), mesenchymal progenitors (*Jag1*, *Tbx2*, *Tbx3*, *Alx4*), connective tissue (*Dcn*, *Kera*, *Col3a1*) and proximal limb (*Tbx15*). Red color scale represents selected markers genes levels of expression from low to high (darker red).

Supplementary figure S7

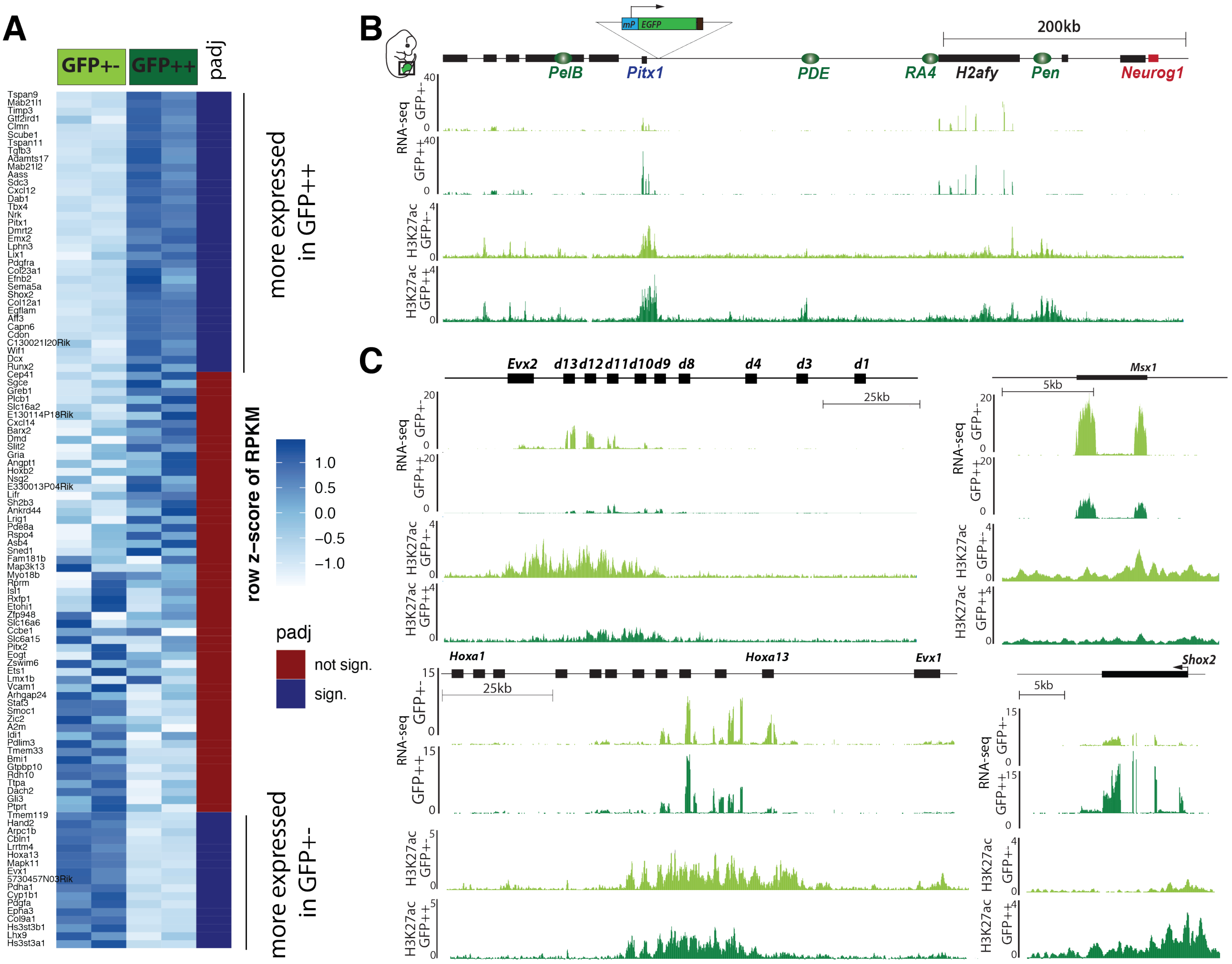

Figure S7: A. Heatmap of *Pitx1*-dependant genes expression as defined in [1] in GFP+- and GFP++ cells. Adjusted p-value (padj) >0.05 is marked in red and <0.05 is marked in blue. **B.** RNA-seq and H3K27ac ChIP-seq tracks in intermediate- and high-expressing hindlimb cell population at the *Pitx1* cluster. **C.** RNA-seq and H3K27ac ChIP-seq tracks in intermediate- and high-expressing hindlimb cell population at the *HoxA and HoxD* cluster as well as at the *Msx1* and *Shox2* locus.

Supplementary figure S8

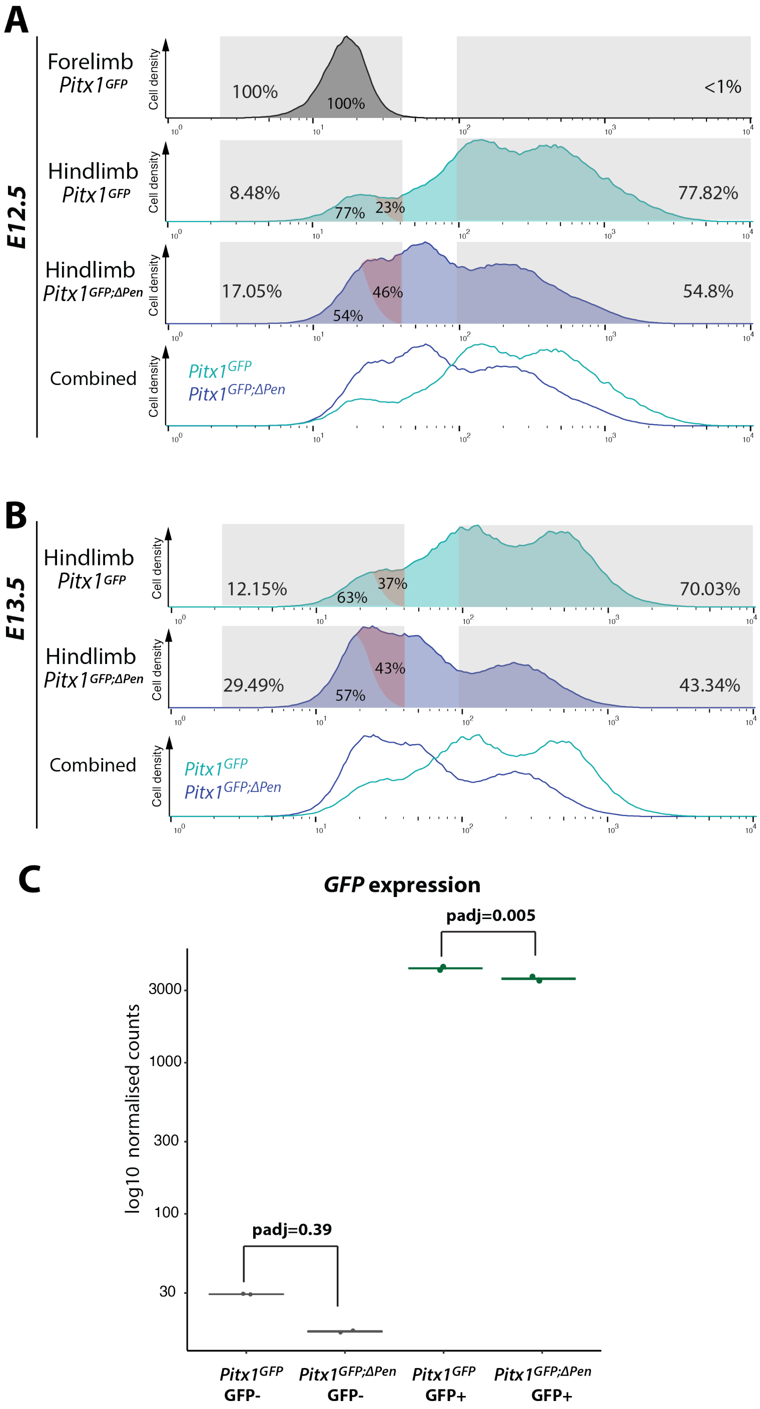

Figure S8: A. Comparison of FACS profile in wildtype E12.5 fore- and hindlimb and in *Pitx1*$^{Pen-/Pen-}$ hindlimb. Note the increase in the negative cell fraction that includes an increase in non- and low cells. In the negative fraction, the low-expressing cells (red shadowed) increased their proportion with respect to non-expressing cells from 23% in wildtype to 46% in mutant (according to surface ratio). **B.** Comparison of FACS profile in wildtype and *Pitx1*$^{Pen-/Pen-}$ E13.5 hindlimbs. In the negative fraction, the low-expressing cells (red shadowed) increased their proportion with respect to non-expressing from 37% in wildtype to 43% in mutant (according to surface ratio). **C.** Normalised counts of *Pitx1* expression in GFP-: *Pitx1*^{GFP} vs *Pitx1*$^{GFP;\Delta Pen}$ and GFP+: *Pitx1*^{GFP} vs *Pitx1*$^{GFP;\Delta Pen}$. Note that there is no change of EGFP expression in GFP- cells and a slight reduction of EGFP expression in GFP+ cells following the deletion of the *Pen* enhancer. Averages are represented by a horizontal bar. Adjusted p-values (padj) of differential gene expression are computed using the Wald-test and Benjamini-Hochberg multiple testing correction as implemented in the Deseq2 tool (Supplementary Dataset S6, S7 and Source Data).

Supplementary figure S9

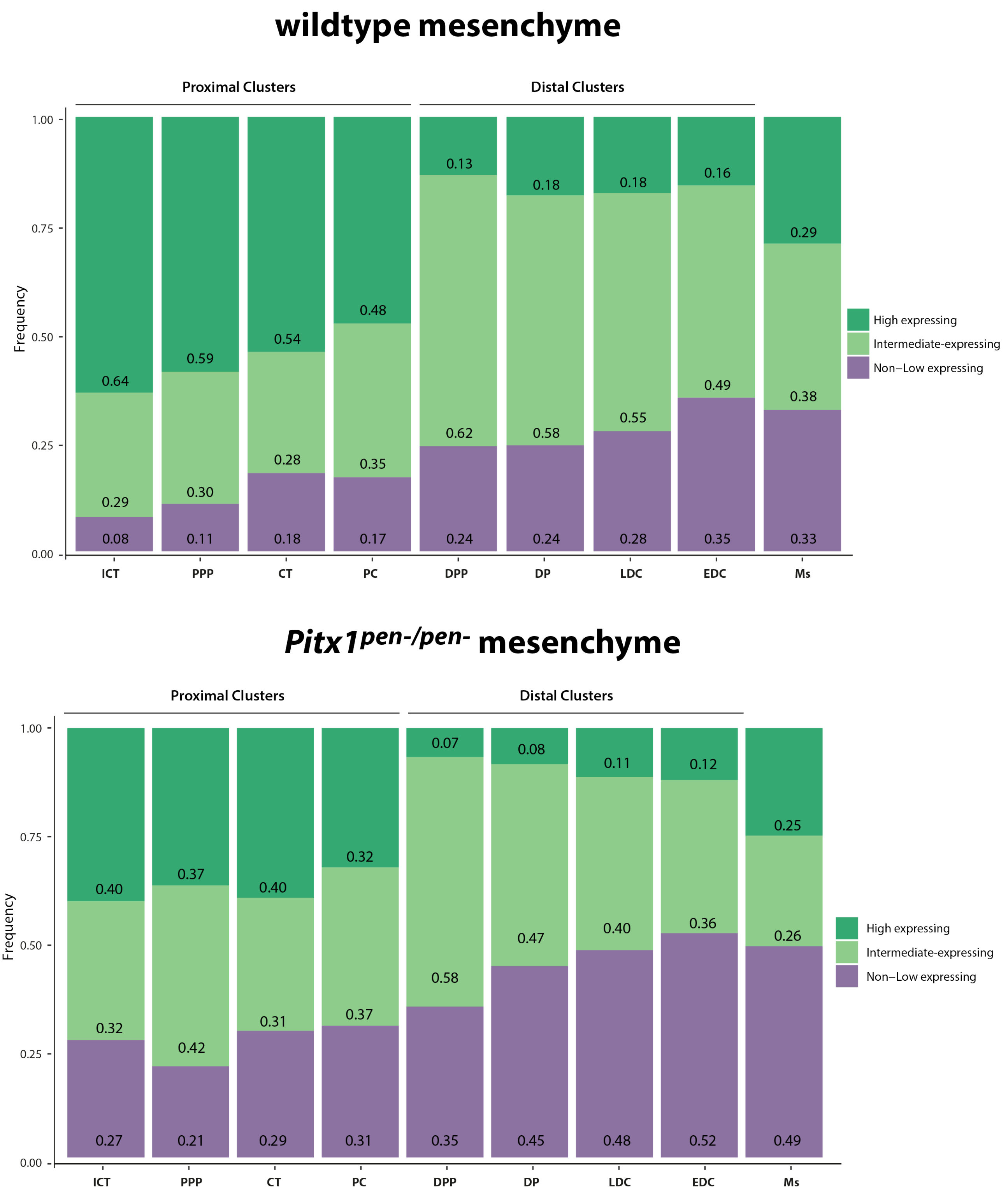

Figure S9: Proportion of *non/low-*, *intermediate* and *high-Pitx1* expressing cells in all mesenchymal cell clusters of wildtype and *Pen* deleted hindlimbs.

Supplementary figure S10

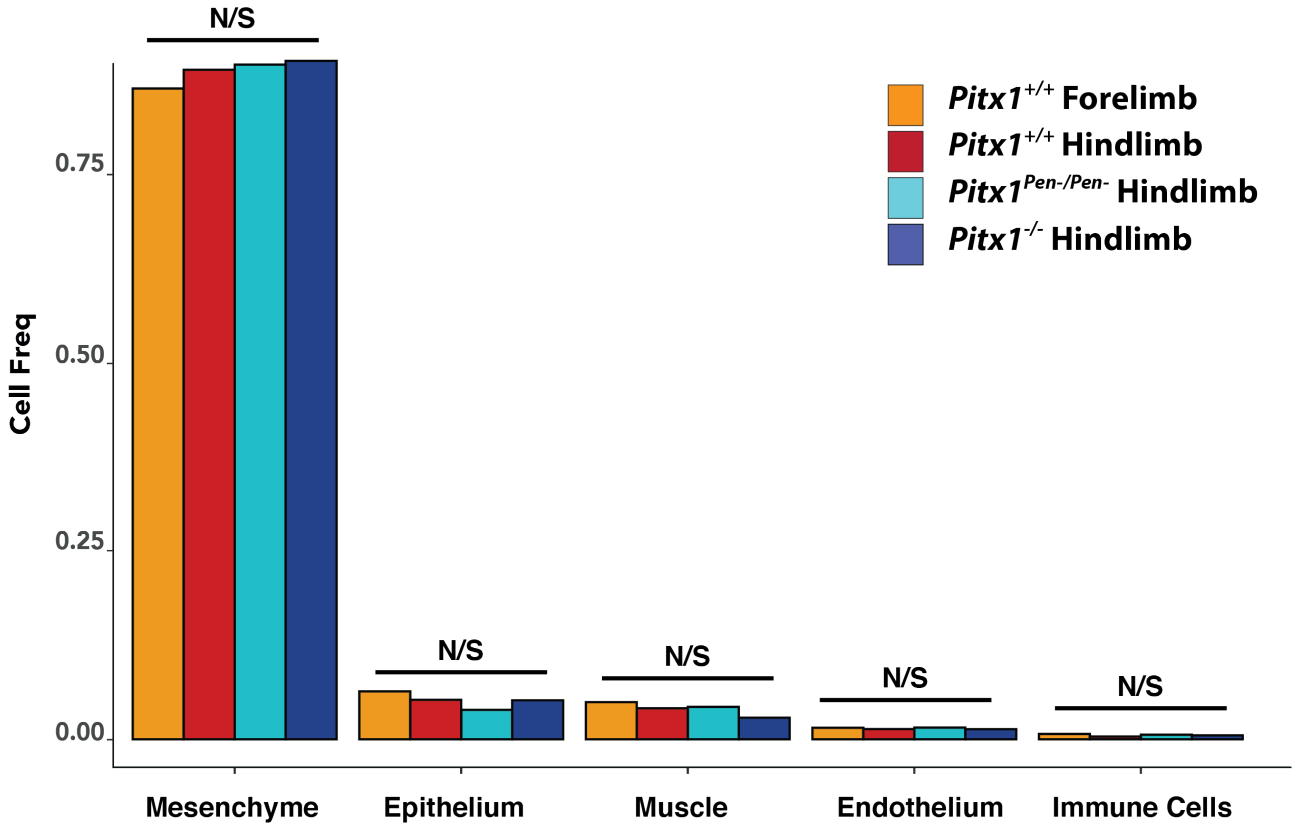

Figure S10: Quantification of satellite cell types (Epithelum, Muscle, Endothelium and Immune Cells) and mesenchyme proportions across conditions. N/S indicates p-value <0.05 for changes in cell proportion. P values were calculated pairwise between wildtype and mutant hindlimbs (See Source Data) using Differential Proportion Analysis in R[2].

Supplementary figure S11

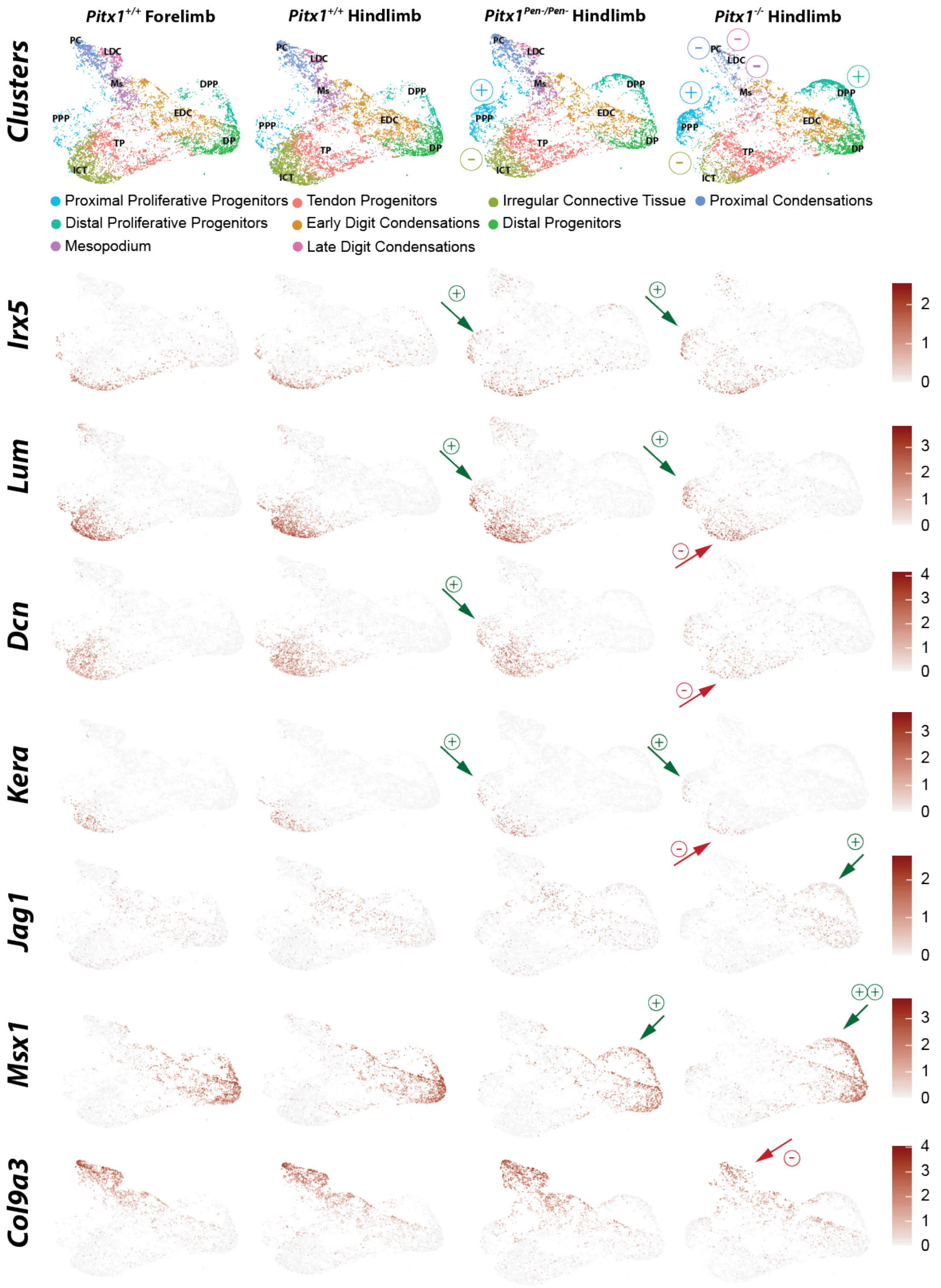

Figure S11: UMAPs of different marker genes in distinct datasets. Note the increased in cell proportion positive for *Irx5* (progenitor marker) and *Lum*, *Kera*, *Dcn* (ICT markers) in the proximal proliferative progenitor cluster (PPP) in *Pitx1*$^{Pen-/Pen-}$ and *Pitx1*$^{-/-}$hindlimbs. Note the loss of cells positive for *Lum*, *Dcn* and *Kera* in *Pitx1*$^{-/-}$hindlimb irregular connective tissue (ICT) cluster. Also note the increase of *Jag1 and Msx1* positive cells in *Pitx1*$^{-/-}$ distal proliferative progenitors (DPP) indicating a higher fraction of progenitor cells. Finally, note the loss differentiated *Col9a3* positive cells in *Pitx1*$^{-/-}$ hindlimbs. Red color scale bar represents selected markers genes levels of expression from low to high (darker red).

Supplementary figure S12

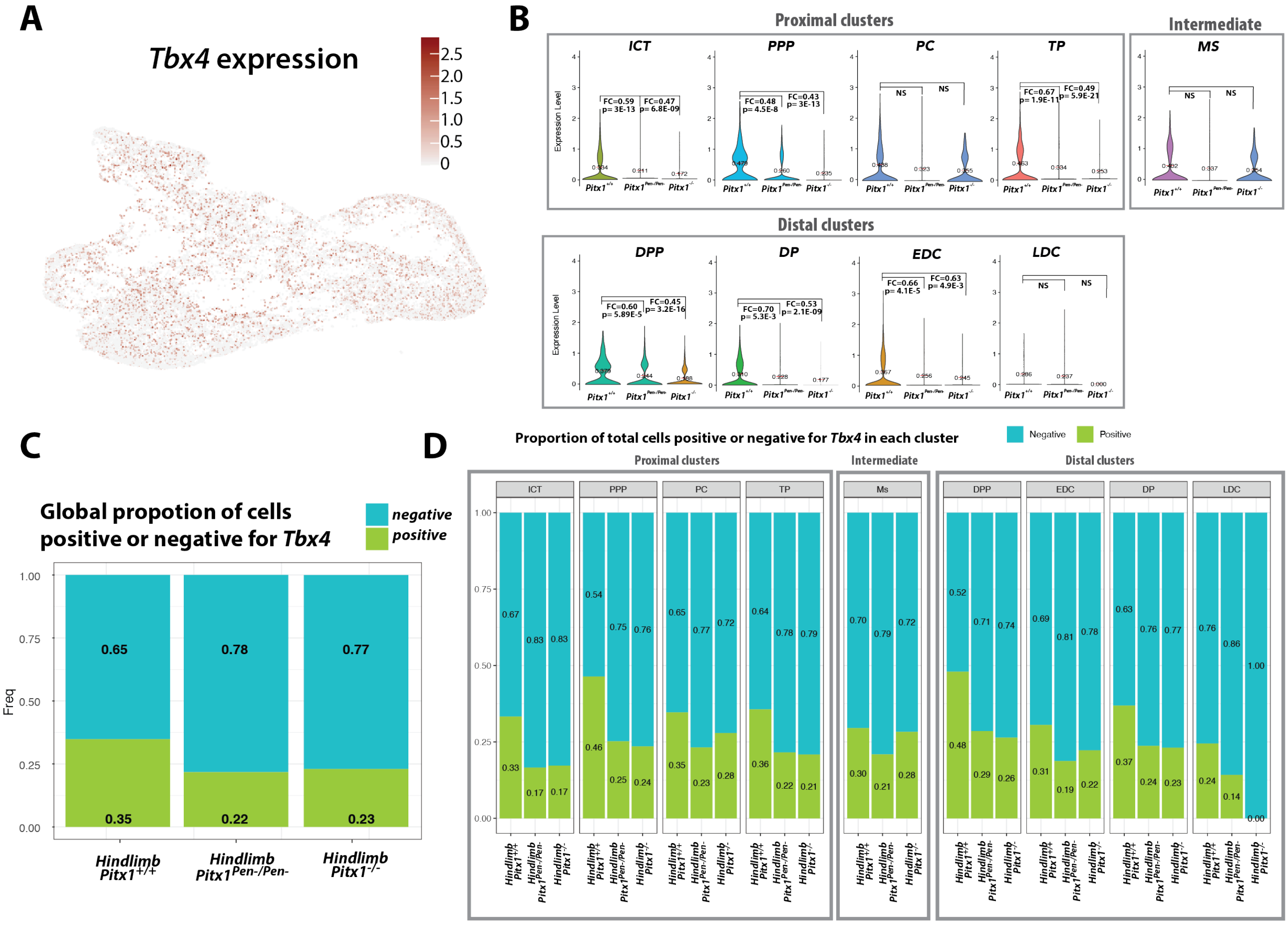

Figure S12: Effect of *Pitx1*$^{-/-}$ and *Pitx1*$^{Pen-/Pen-}$ on *Tbx4* hindlimb expression. A. UMAP of *Tbx4* expression within the mesenchymal cluster. Red color scale bar represents *Tbx4* levels of expression from low to high (darker red). **B.** Violin plots of *Tbx4* expression in all mesenchymal clusters in wildtype, *Pitx1*$^{-/-}$ and *Pitx1*$^{Pen-/Pen-}$ hindlimbs. Adjusted p-values (p) shown in the figure were calculated by Wilcoxon Ranks Sum test using the FindMarkers function from Seurat R Package (Supplementary Dataset S5). **C.** Global proportion of *Tbx4* expressing and non-expressing cells across the mesenchyme in wildtype, *Pitx1*$^{-/-}$ and *Pitx1*$^{Pen-/Pen-}$ hindlimbs. **D.** Proportion of *Tbx4* expressing and non-expressing cells across all mesenchymal clusters in wildtype, *Pitx1*$^{-/-}$ and *Pitx1*$^{Pen-/Pen-}$ hindlimbs.

Supplementary figure S13

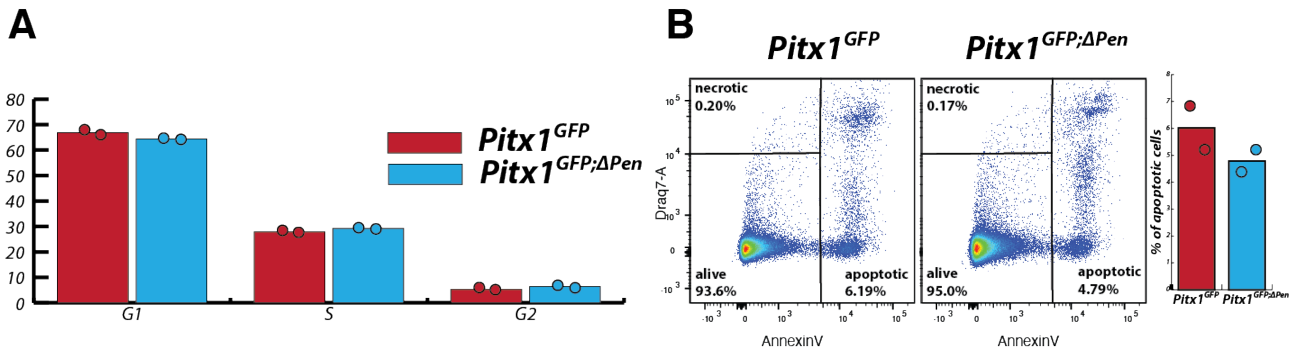

Figure S13: Effect of *Pen* enhancer deletion on cell proliferation and apoptosis. A. Comparison of *Pitx1*^{GFP} and *Pitx1*$^{GFP;\Delta Pen}$ proliferation rate using Hoechst staining (N=2) (See Source Data). Note the absence of important changes in the relative proportion of G1, S and G2 cells. **B.** Comparison of *Pitx1*^{GFP} and *Pitx1*$^{GFP;\Delta Pen}$ apoptotic rate using AnnexinV assay (N=2) (See Source Data). Note the absence of important relative proportion change of apoptotic cells.

Supplementary figure S14

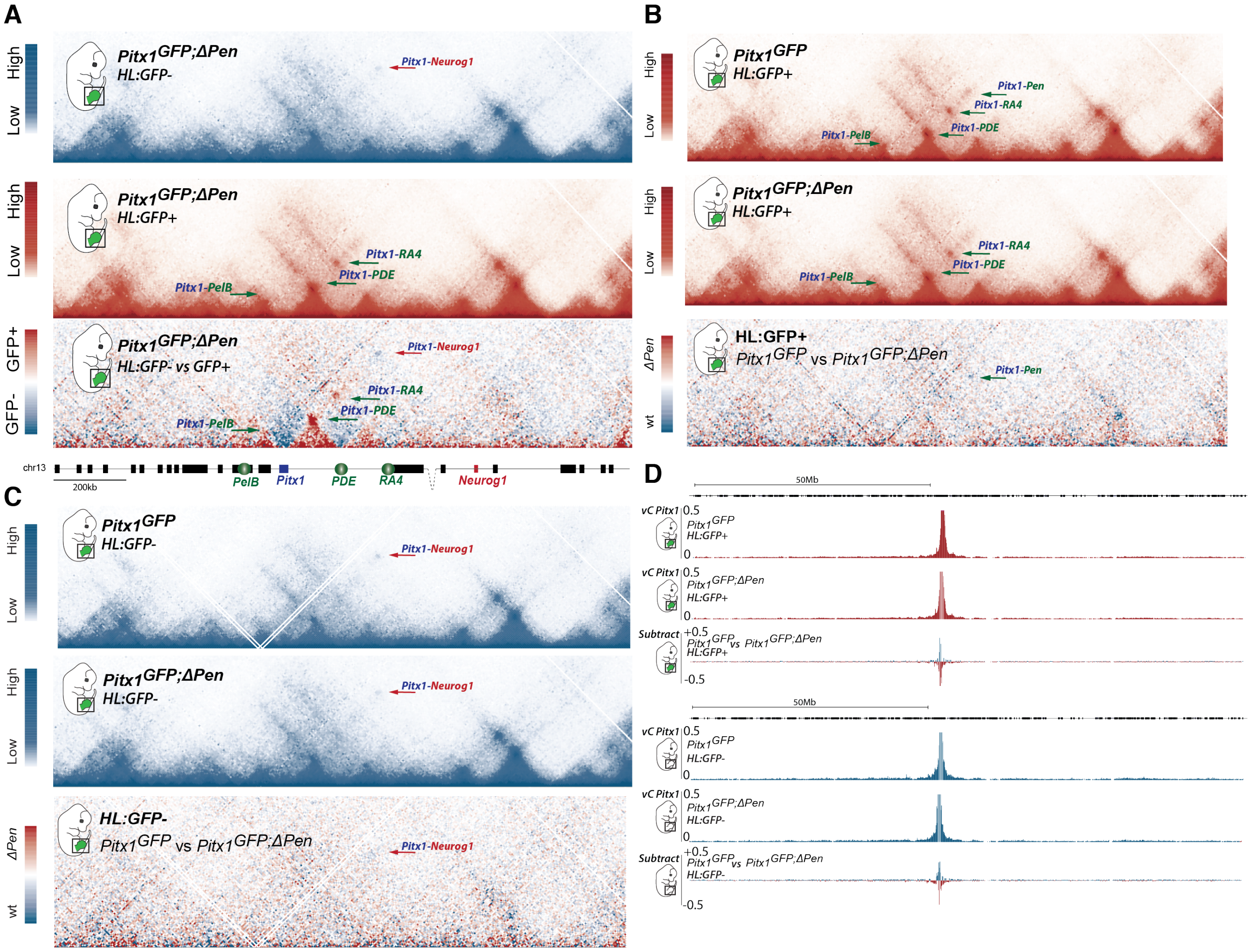

Figure S14: A. C-HiC in GFP- (upper panel) and GFP+ (middle panel) sorted hindlimb cells in *Pitx1*$^{GFP;\Delta Pen}$ hindlimbs. GFP- interactions are displayed in blue and *Pitx1*$^{GFP;\Delta Pen}$ GFP+ interaction in red. Darker red bins represent more frequent contacts as represented by scaled bars on the left. The lower panel represents a subtraction between the two upper ones. GFP+ preferential interactions are displayed in red and GFP- preferential interactions in blue. **B.** C-HiC of *Pitx1*^{GFP} GFP+ (upper panel) and *Pitx1*$^{GFP;\Delta Pen}$ GFP+ (middle panel) hindlimb cells. Darker red bins represent more frequent contacts as represented by scaled bars on the left. The lower panel represents subtraction of these two maps. *Pitx1*$^{GFP;\Delta Pen}$ GFP+ preferential interactions are displayed in red and *Pitx1*^{GFP} GFP+ preferential interactions in blue. **C.** C-HiC of *Pitx1*^{GFP} GFP- (upper panel) and *Pitx1*$^{GFP;\Delta Pen}$ GFP- (middle panel) hindlimb cells. Darker blue bins represent more frequent contacts as represented by scaled bars on the left. The lower panel represents subtraction of these two maps. *Pitx1*$^{GFP;\Delta Pen}$ GFP- preferential interactions are displayed in red and *Pitx1*^{GFP} GFP- preferential interactions in blue. **D.** Virtual 4C from the *Pitx1* viewpoint toward the whole chromosome 13 in GFP+ (two upper red tracks and subtraction track below) and GFP- (two lower blue tracks and subtraction track below). Note the absence of change in both subtraction tracks outside of the *Pitx1* locus.

Supplementary Table S1

mutant	sgRNA1	sgRNA2	genomic location	
PItx1 -GFP	GAAACCGGGAGACGGTGATC	N/A	chr13:55935371-55935390	N/A
Pitx1- del*Pen*	GCCTAGTGGAGGCGCGGCTT	CGTGCTATCGAGGGACTAAT	centromeric - chr13:56260947-56260966	telomeric- chr13:56262116-56262135
Pitx1- del	TATCCAGGCAGTTTCACCCC	GCTGTAGTTAAAGGAAGCTGGG	centromeric - chr13:55923035-55923054	telomeric - chr13:55939227-55939248
mutant	genotyping primer pair 1 Forward	genotyping primer pair 1 Reverse	genotyping primer pair 2 Forward	genotyping primer pair 2 Reverse
PItx1 -GFP	GTGTACGGGTGGACACACAC	CGCTGTTTGGTCTGCTTTCT	GAACTTCAGGGTCAGCTTGC	TTGCGTGCTAGGATGTAACG
Pitx1- del*Pen*	GTTGTGTTCACGGTCAGTGCTGTG[illegible]	CCCTTGGAGCTGCTCTCAAGGTG	N/A	N/A
Pitx1- del	TATCCAGGCAGTTTCACCCC	GCTGTAGTTAAAGGAAGCTGGG	TGGTCCCCAGTACTTGCCTA	GCTGTAGTTAAAGGAAGCTGGG

Table S1: sgRNAs and genotyping oligonucleotides used in this study and relative genomic location into NCBI37/mm9 reference genome.

Supplementary Info References

1 Nemec, S. *et al.* Pitx1 directly modulates the core limb development program to implement hindlimb identity. *Development*, doi:10.1242/dev.154864 (2017).

2 Farbehi, N. *et al.* Single-cell expression profiling reveals dynamic flux of cardiac stromal, vascular and immune cells in health and injury. *eLife* **8**, doi:10.7554/eLife.43882 (2019).
