# Supplementary material for: Cell-specific alterations in Pitx1 regulatory landscape activation caused by the loss of a single enhancer
Source: Nat Commun. 2021 Dec 13;12:7235. doi: 10.1038/s41467-021-27492-1 (PMC8668926; doi:10.1038/s41467-021-27492-1)
Supplement: Supplementary file 3 — Description of Additional Supplementary Files [file 41467_2021_27492_MOESM3_ESM.docx]

**Legends to supplementary datasets and video**

**Supplementary Dataset S1:** Marker genes for single-cell clusters. Marker genes, avg.logFC, cell percentage (pct.1 = cells in a given cluster, pct2= all the remaining cells) and p value adj. were calculated using the FindMarkers (from the Seurat R package) using Wilcoxon Ranks Sum test.

**Supplementary Dataset S2:** DeSeq2 analysis of *Pitx1^+/+^* vs *Pitx1^GFP^* hindlimbs. Positive FC indicate enrichment in *Pitx1^GFP^* and negative FC indicate enrichment in *Pitx1^+/+^* hindlimbs. Adjusted p-values (padj) of differential gene expression are computed using the Wald-test and Benjamini-Hochberg multiple testing correction as implemented in the Deseq2 tool.

**Supplementary Dataset S3:** DeSeq2 analysis of GFP- vs GFP+ *Pitx1^GFP^* hindlimbs. Positive FC indicate enrichment in GFP+ cells and negative FC indicate enrichment in GFP- cells. Adjusted p-values (padj) of differential gene expression are computed using the Wald-test and Benjamini-Hochberg multiple testing correction as implemented in the Deseq2 tool.

**Supplementary Dataset S4:** DeSeq2 analysis of GFP- (non/low) vs GFP+- (intermediate). Positive FC indicate enrichment in GFP+- cells and negative FC enrichment in GFP- cells. DeSeq2 analysis of GFP+- (intermediate) vs GFP++ (high) transcriptomes. Positive FC indicate enrichment in GFP++ cells and negative FC enrichment in GFP+- cells. Adjusted p-values (padj) of differential gene expression are computed using the Wald-test and Benjamini-Hochberg multiple testing correction as implemented in the Deseq2 tool.

**Supplementary Dataset S5:** Differential gene expression in the mesenchyme and all of its clusters comparing *Pitx1^+/+^* vs *Pitx1^Pen-/Pen-^* single-cell datasets. Foldchange and p values are calculated using the FindMarkers (from the Seurat R package) function that performs a Wilcoxon Ranks Sum test.

**Supplementary Dataset S6:**DeSeq2 analysis of *Pitx1^GFP^* vs *Pitx1^GFP;ΔPen^*  *GFP-* hindlimb cells. Positive FC indicates enrichment in GFP- cells of *Pitx1^GFP;ΔPen^*  hindlimbs and negative FC indicates depletion in GFP- cells from *Pitx1^GFP^* hindlimbs. Adjusted p-values (padj) of differential gene expression are computed using the Wald-test and Benjamini-Hochberg multiple testing correction as implemented in the Deseq2 tool.

**Supplementary Dataset S7:** DeSeq2 analysis of *Pitx1^GFP^* vs *Pitx1^GFP;ΔPen^*  *GFP+* hindlimb cells. Positive FC indicates enrichment in GFP+ cells of *Pitx1^GFP;ΔPen^*  hindlimbs and negative FC indicates depletion in GFP+ cells from *Pitx1^GFP^* hindlimbs. Adjusted p-values (padj) of differential gene expression are computed using the Wald-test and Benjamini-Hochberg multiple testing correction as implemented in the Deseq2 tool.

**Supplementary Video S1:** 3D reconstruction of a *Pitx1^GFP^* E12.5 embryo. Scale bar = 2mm.
