# Supplementary material for: Cell-specific alterations in Pitx1 regulatory landscape activation caused by the loss of a single enhancer
Source: Nat Commun. 2021 Dec 13;12:7235. doi: 10.1038/s41467-021-27492-1 (PMC8668926; doi:10.1038/s41467-021-27492-1)
Supplement: Supplementary file 12 — Reporting Summary [file 41467_2021_27492_MOESM12_ESM.pdf]

nature portfolio

Corresponding author(s): Guillaume Andrey

Last updated by author(s): 18.11.2021

Reporting Summary

Nature Portfolio wishes to improve the reproducibility of the work that we publish. This form provides structure for consistency and transparency in reporting. For further information on Nature Portfolio policies, see our Editorial Policies and the Editorial Policy Checklist.

Statistics

For all statistical analyses, confirm that the following items are present in the figure legend, table legend, main text, or Methods section.

n/a	Confirmed	
☐	☒	The exact sample size (*n*) for each experimental group/condition, given as a discrete number and unit of measurement
☒	☐	A statement on whether measurements were taken from distinct samples or whether the same sample was measured repeatedly
☐	☒	The statistical test(s) used AND whether they are one- or two-sided<br>*Only common tests should be described solely by name; describe more complex techniques in the Methods section.*
☒	☐	A description of all covariates tested
☒	☐	A description of any assumptions or corrections, such as tests of normality and adjustment for multiple comparisons
☐	☒	A full description of the statistical parameters including central tendency (e.g. means) or other basic estimates (e.g. regression coefficient) AND variation (e.g. standard deviation) or associated estimates of uncertainty (e.g. confidence intervals)
☐	☒	For null hypothesis testing, the test statistic (e.g. *F*, *t*, *r*) with confidence intervals, effect sizes, degrees of freedom and *P* value noted<br>*Give P values as exact values whenever suitable.*
☒	☐	For Bayesian analysis, information on the choice of priors and Markov chain Monte Carlo settings
☒	☐	For hierarchical and complex designs, identification of the appropriate level for tests and full reporting of outcomes
☒	☐	Estimates of effect sizes (e.g. Cohen's *d*, Pearson's *r*), indicating how they were calculated

Our web collection on statistics for biologists contains articles on many of the points above.

Software and code

Policy information about availability of computer code

Data collection: No commercial, open source or custom software/code was used to collect the data in this study

Data analysis:

cHi-C:
Paired-end reads from sequencing were mapped to the reference genome NCBI37/mm9 using Bowtie2 version 2.3.4.2 (Langmead and Salzberg 2012) and further filtered and deduplicated using HiCUP version 0.6.1. When replicates were available, these were pooled through catenation (-cat in Python 2.7.11) before HiCUP analysis. Valid and unique di-tags were filtered and further processed with Juicer tools version 1.9.9 to produce binned contact maps from valid read pairs with MAPQ ≥ 30 and maps were normalized using Knights and Ruiz matrix balancing, considering only the genomic region chr13: 54,000,001-57,300,000 (Knight and Ruiz 2013; Wingett et al. 2015; Durand et al. 2016). After KR normalization, maps were exported at 5kb resolution.

ChIP-seq:
Single-end reads were mapped to the reference genome NCBI37/mm9 using Bowtie2 version 2.3.4.2 (Langmead and Salzberg 2012), filtered for mapping quality q ≥ 25 and duplicates were removed with SAMtools 1.9. Reads were extended to 250 bp and scaled (1 million/total of unique reads) to produce coverage tracks using genomecov of BEDTools/2.28.0-fecbf4e3. BigWig files were produced using bedGraphToBigWig version 4 and visualized in the UCSC genome browser.

FACS
Profiles were analysed using FlowJoTM v10.6.1. Replicate analysis and presentation were performed using Microsoft Excel for Mac 2011 (Version 14.7.2)

RNA-seq
Single-end reads were mapped to the mm9 reference genome using STAR mapper version 2.5.2a with default settings. Further processing was done according to (Paliou et al. 2019). BigWig files were visualized in the UCSC genome browser. Counting was done using R version 3.6.2 and

differential expression was analyzed through the "DEseq2" R package version 3.14.

Single Cell:
A wide range of softwares were used for single cell analyses (see below), yet for more information about the used parameters please check the material and methods section of the manuscript.
Cell Ranger software v3.0.2
velocyto.py tool v 0.17.17
Seurat package v 3.2.0
Seurat Wrappers package v0.2.0
Scvelo v 0.2.2

For manuscripts utilizing custom algorithms or software that are central to the research but not yet described in published literature, software must be made available to editors and reviewers. We strongly encourage code deposition in a community repository (e.g. GitHub). See the Nature Portfolio guidelines for submitting code & software for further information.

Data

Policy information about availability of data

All manuscripts must include a data availability statement. This statement should provide the following information, where applicable:

- Accession codes, unique identifiers, or web links for publicly available datasets
- A description of any restrictions on data availability
- For clinical datasets or third party data, please ensure that the statement adheres to our policy

Sequencing data are available at the GEO repository under the accession number GSE168633.

Field-specific reporting

Please select the one below that is the best fit for your research. If you are not sure, read the appropriate sections before making your selection.

☒ Life sciences ☐ Behavioural & social sciences ☐ Ecological, evolutionary & environmental sciences

For a reference copy of the document with all sections, see nature.com/documents/nr-reporting-summary-flat.pdf

Life sciences study design

All studies must disclose on these points even when the disclosure is negative.

Sample size	Sample size is 2-3 replicates of 2-8 limb pairs for scRNA-seq, FACS analyses (AnnexinV and Hoechst), bulk RNA-seq and cell-sorted RNA-seq (GFP+/GFP-; GFP++/GFP+-). Cell-sorted cHi-C and H3K27ac ChIP-seq experiments were performed from singlicates of 7-10 FACS-sorted pooled limb pairs. These are commonly accepted sample size for these types of experiments. The minimal number of sorted or unsorted cells for all experiments, and thus the number of pooled limb pairs, are determined by the type of experiments (RNA-seq: 150K cells, FACS: 200K cells, scRNA-seq: 20K cells, c-Hi-C: 1M cells and ChIP-seq: 500K cells). Therefore, each replicate result is an average of measurements across many samples. Finally, the number of samples per experiment is limited by the cost, especially for single-cell experiments. For WISH and fluorescence microscopy experiments, at least three E12.5 embryos were checked.
Data exclusions	No data was excluded from this study
Replication	Experiments were performed in replicates when the results were likely to have a significant impact on the interpretation of the results. RNA-seq were performed in duplicates, with the exception of bulk wildtype and GFP-sensor hindlimbs which were done in biological triplicates. All RNA-seq replication attempts were successful. For scRNA-seq experiments, two replicates were used for every condition and all of them were successful. FACS analyses were done in duplicates or triplicates as indicated in the corresponding figures and all of these replication attempts were successful. H3K27ac ChIP-seq and Capture Hi-C experiments were performed in singlicates of 7-10 FACS-sorted pooled limb pairs. WISH and fluorescence microscopy experiments were imaged in triplicates and all of these attempts were successful.
Randomization	In this work, it was necessary to know the genotypes and types of analyzed cells (GFP-/GFP+ ; GFP++/GFP+-) as they needed to be pooled prior to processing for experiments.
Blinding	Investigators were not blinded since micro-dissection of mouse embryos and further analyses require knowledge about the genotype and type of cells (GFP-/GFP+ ; GFP++/GFP+-) at hand.

Reporting for specific materials, systems and methods

We require information from authors about some types of materials, experimental systems and methods used in many studies. Here, indicate whether each material, system or method listed is relevant to your study. If you are not sure if a list item applies to your research, read the appropriate section before selecting a response.

Materials & experimental systems

n/a	Involved in the study
☐	☒ Antibodies
☐	☒ Eukaryotic cell lines
☒	☐ Palaeontology and archaeology
☐	☒ Animals and other organisms
☒	☐ Human research participants
☒	☐ Clinical data
☒	☐ Dual use research of concern

Methods

n/a	Involved in the study
☐	☒ ChIP-seq
☐	☒ Flow cytometry
☒	☐ MRI-based neuroimaging

Antibodies

Antibodies used	Polyclonal rabbit anti-H3K27ac antibody from Diagenode, Cat-No. c15410174, was used at a dilution of 1/500.
Validation	The antibody was validated for ChIP usage on the manufacturer's website: https://www.diagenode.com/en/p/h3k27ac-polyclonal-antibody-classic-50-mg-42-ml. Moreover, we extensively used the same antibody in a prior study and observed a high reproducibility (Andrey et al., 2017).

Eukaryotic cell lines

Policy information about cell lines

Cell line source(s)	Male G4 mouse ESCs (Jorge et al., 2007)
Authentication	Genetically modified pluripotent ESCs were authenticated by the production of embryos through tetraploid aggregations (Artus and Hadjantonakis 2011) and further genotyping confirmed the presence of the desired mutations in the cells
Mycoplasma contamination	All cell lines tested negative for mycoplasma contamination.
Commonly misidentified lines (See ICLAC register)	No commonly misidentied cell lines were used.

Animals and other organisms

Policy information about studies involving animals; ARRIVE guidelines recommended for reporting animal research

Laboratory animals	Embryos were generated from male G4 mouse ESCs (Jorge et al., 2007) via tetraploid complementation (Artus and Hadjantonakis 2011). Donor tetraploid embryos were provided from in vitro fertilisation using c57bl6J x B6D2F1 backgrounds. Aggregated embryos were transferred into CD1 foster females and micro-dissected at embryonic stage E12.5.
Wild animals	There was no wild animals in this study
Field-collected samples	There was no field collected animals in this study
Ethics oversight	All animal procedures were in accordance with institutional, state, and government regulations (Canton de Genève authorisation: GE/89/19).

Note that full information on the approval of the study protocol must also be provided in the manuscript.

ChIP-seq

Data deposition

☒ Confirm that both raw and final processed data have been deposited in a public database such as GEO.

☒ Confirm that you have deposited or provided access to graph files (e.g. BED files) for the called peaks.

Data access links *May remain private before publication.*	Datasets are available through the GEO repository under the accession number GSE168633, the ChIP-seq dataset in particular is available under the accession number (GSE168631): https://www.ncbi.nlm.nih.gov/geo/query/acc.cgi?acc=GSE168631
Files in database submission	H3K27ac-HL-E125-delPen-GFPn_R1_S4_L004_R1_001.fastq.gz<br>H3K27ac-HL-E125-delPen-GFPp_R1_S3_L004_R1_001.fastq.gz<br>H3K27ac-HL-E125-WT-GFPn_R1_S1_L001_R1_001.fastq.gz<br>H3K27ac-HL-E125-WT-GFPp-R1_S2_L004_R1_001.fastq.gz<br>H3K27ac-HL-E125-WT-GFPpMild_S14_R1_001.fastq.gz<br>H3K27ac-HL-E125-WT-GFPpHigh_S13_R1_001.fastq.gz

H3K27ac-HL-E125-delPen-GFPn_R1.bw
H3K27ac-HL-E125-delPen-GFPp_R1.bw
H3K27ac-HL-E125-WT-GFPn_R1.bw
H3K27ac-HL-E125-WT-GFPp_R1.bw
H3K27ac-HL-E125-WT-GFPpHigh_R1_001.bw
H3K27ac-HL-E125-WT-GFPpMild_R1_001.bw

Genome browser session (e.g. UCSC)	Not applicable

Methodology

Replicates	ChIP-seq were performed in singlicates from FACS-sorted cells from 7-10 pooled pairs of limb buds.
Sequencing depth	H3K27ac-HL-E125-delPen-GFPp_R1_S3_L004_R1_001.fastq.gz: 103971210<br>H3K27ac-HL-E125-delPen-GFPn_R1_S4_L004_R1_001.fastq.gz: 83232922<br>H3K27ac-HL-E125-WT-GFPn_R1_S1_L001_R1_001.fastq.gz: 104209261<br>H3K27ac-HL-E125-WT-GFPp-R1_S2_L004_R1_001.fastq.gz: 103420803<br>H3K27ac-HL-E125-WT-GFPpHigh_S13_R1_001.fastq.gz: 44620478<br>H3K27ac-HL-E125-WT-GFPpMild_S14_R1_001.fastq.gz: 42234088
Antibodies	H3K27ac (Diagenode C15410174)
Peak calling parameters	There was no peak calling used in this project
Data quality	Enrichment was validated using spp cross-correlation plots
Software	Bowtie2/2.3.4.2<br>SAMtools/1.9<br>BEDTools/2.28.0-fecbf4e3<br>bedGraphToBigWig version 4

Flow Cytometry

Plots

Confirm that:

- [x] The axis labels state the marker and fluorochrome used (e.g. CD4-FITC).
- [x] The axis scales are clearly visible. Include numbers along axes only for bottom left plot of group (a 'group' is an analysis of identical markers).
- [x] All plots are contour plots with outliers or pseudocolor plots.
- [x] A numerical value for number of cells or percentage (with statistics) is provided.

Methodology

Sample preparation	Forelimb and hindlimb buds were dissected in cold PBS solution. After PBS removal, a single cell suspension was achieved by incubating the limb buds in 400uL Trypsin-EDTA (Thermo Fischer Scientific, 25300062) for 12' at 37°C in a Thermomixer with a resuspension step at the 6' mark. After blocking with one volume of 5% BSA (Sigma Aldrich, A7906-100G), cells were passed through a 40µm cell strainer for further tissue disruption and another volume of 5% BSA was added to the cell strainer to pass leftover cells. Cells were then centrifuged at 400g for 5' at 4°C and, after discarding the supernatant, they were resuspended in 1% BSA for cell sorting. 5mM of NaButyrate were added to the BSA when planning for subsequent fixation for H3K27Ac-ChIP.
Instrument	Cell populations were isolated using fluorescent-activated cell sorting (FACS) using the Beckman Coulter MoFlo Astrios.
Software	Flow cytometry analyses to obtain EGFP histograms were performed with the FlowJoTM Software (version 10.6.1).
Cell population abundance	Borders to determine population abundance are displayed in main and supplemental figures
Gating strategy	Initial FSC/SCC was set between 30/40 and 210/240 to exclude debris. After removal of dead cells with Draq7 dye and removal of doublets, following standard protocol, cells were gated for sorting as can be seen in FigS1A. As a control, a non-GFP expressing tissue (forelimbs isolated from the same E12.5 embryos) was used to determine the gating of the GFP-fraction of the samples to sort. When multiple cell sortings were needed, gating was done in accordance to previous samples to ensure non-variability in GFP intensity.

- [x] Tick this box to confirm that a figure exemplifying the gating strategy is provided in the Supplementary Information.
